# Proactive and reactive cognitive control and dorsolateral prefrontal cortex dysfunction in first episode schizophrenia^☆^

**DOI:** 10.1016/j.nicl.2013.04.010

**Published:** 2013-04-22

**Authors:** Tyler A. Lesh, Andrew J. Westphal, Tara A. Niendam, Jong H. Yoon, Michael J. Minzenberg, J. Daniel Ragland, Marjorie Solomon, Cameron S. Carter

**Affiliations:** a Department of Psychiatry, University of California, Davis, USA; b Department of Psychology, University of California, Los Angeles, USA; c MIND Institute, University of California, Davis, USA; d Department of Psychology, University of California, Davis, USA

**Keywords:** Cognitive control, Schizophrenia, DLPFC, Stroop, AX-CPT, fMRI

## Abstract

Cognitive control deficits have been consistently documented in patients with schizophrenia. Recent work in cognitive neuroscience has hypothesized a distinction between two theoretically separable modes of cognitive control—reactive and proactive. However, it remains unclear the extent to which these processes are uniquely associated with dysfunctional neural recruitment in individuals with schizophrenia. This functional magnetic resonance imaging (fMRI) study utilized the color word Stroop task and AX Continuous Performance Task (AX-CPT) to tap reactive and proactive control processes, respectively, in a sample of 54 healthy controls and 43 patients with first episode schizophrenia. Healthy controls demonstrated robust dorsolateral prefrontal, anterior cingulate, and parietal cortex activity on both tasks. In contrast, patients with schizophrenia did not show any significant activation during proactive control, while showing activation similar to control subjects during reactive control. Critically, an interaction analysis showed that the degree to which prefrontal activity was reduced in patients versus controls depended on the type of control process engaged. Controls showed increased dorsolateral prefrontal cortex (DLPFC) and parietal activity in the proactive compared to the reactive control task, whereas patients with schizophrenia did not demonstrate this increase. Additionally, patients' DLPFC activity and performance during proactive control was associated with disorganization symptoms, while no reactive control measures showed this association. Proactive control processes and concomitant dysfunctional recruitment of DLPFC represent robust features of schizophrenia that are also directly associated with symptoms of disorganization.

## Highlights

• Cognitive control and fronto-parietal recruitment are disrupted in schizophrenia.
• Schizophrenia patients show hypoactivation during proactive not reactive control.
• DLPFC activity was associated with disorganization only in proactive control.
• Proactive control deficits may reflect a more robust marker of disease pathology.

## 1 Introduction

While decades of research illustrate that schizophrenia is associated with deficits across a wide variety of cognitive domains, including attention, memory, and language, recent theories propose that impaired cognitive control-related dysfunction of the prefrontal cortex may account for many of these findings (Barch and Ceaser, 2012; Lesh et al., 2011). Cognitive control involves online maintenance of goals and task sets to guide adaptive behavior (Miller and Cohen, 2001). An extensive literature suggests that patients with schizophrenia show cognitive control deficits (Cohen and Servan-Schreiber, 1992; Cohen et al., 1996, 1999; Servan-Schreiber et al., 1996) associated with impaired recruitment of the dorsolateral prefrontal cortex (DLPFC) and related circuitry, including the anterior cingulate cortex (ACC) and parietal cortex (Barch et al., 2001; MacDonald and Carter, 2003; MacDonald et al., 2000; Yoon et al., 2008).

Recent work highlights a distinction between proactive and reactive modes of cognitive control, which Braver and colleagues have termed the dual mechanisms of control (DMC) theory (Braver et al., 2007, 2009). Proactive control is conceptualized as maintenance of goal-relevant information to optimally bias attention, perception and response preparation ahead of a cognitively demanding event. In contrast, reactive control reflects transient, ‘on the fly’ engagement of control processes at the onset of challenging task demands. Due to its connectivity with sensorimotor regions, DLPFC plays a central role in the maintenance of goals and rules for action (Asaad et al., 2000; Watanabe, 1990, 1992), which should be reflected in increased DLPFC activity during proactive control. Proactive control may depend more on the ability to mount a sustained pattern of neural activity, a well-characterized aspect of prefrontal cortex that is observed during both physiological recording in non-human primates (Goldman-Rakic, 1995) and fMRI in humans (MacDonald et al., 2000). In contrast, reactive control may be associated with more transient neural engagement of not only DLPFC, but also ACC, which is associated with detecting conflict and recruiting DLPFC engagement on a trial to trial basis (Egner and Hirsch, 2005; Kerns et al., 2005; MacDonald et al., 2000). Edwards et al. (2010) investigated proactive and reactive control processes in patients with schizophrenia using a version of the AX Continuous Performance Task (AX-CPT), in which the cue (proactive control) and probe (reactive control) phases were examined separately. Patients with schizophrenia showed more probe-related activity while controls demonstrated greater cue-related PFC activity, suggesting that patients were relying on reactive control. Further, after training patients to recognize and attend to contextual information, they showed a significant shift in brain activation (i.e., increased cue-related activity and decreased probe-related activity) reflecting changing emphasis from reactive to proactive control.

**Fig. 1:**
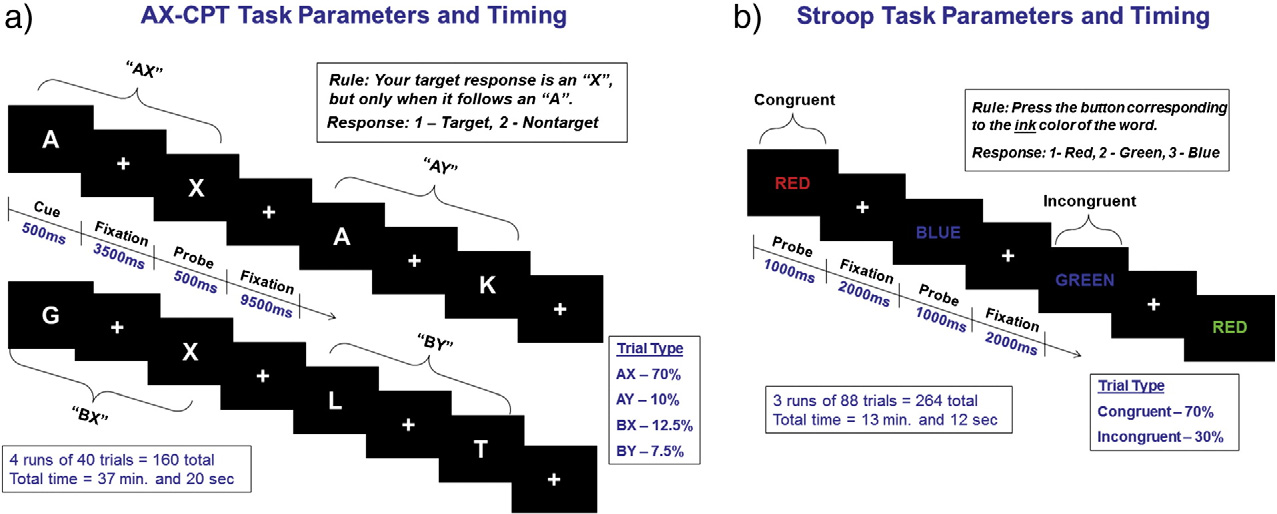
Task parameters for the a) AX-CPT and b) Stroop. Tasks were presented using E-prime software (http://www.pstnet.com/eprime.cfm) running on an IBM-compatible computer. Presentation of stimuli was pseudorandom, and the first two stimuli for each subject were target (AX) trials for the AX-CPT and Congruent trials for the Stroop.

While these data provide evidence for the dissociation between proactive and reactive control, examining these processes within the same task has some limitations. First, reactive control is necessarily linked to contextual processes engaged during the cue, thus the reactive control measure is inherently confounded by the degree to which proactive processes are engaged. This might attenuate reactive activity in controls due to their intact proactive processes. While this may be unavoidable for any task, as individuals often engage context maintenance to guide responding, this limitation may be partially mitigated by choosing tasks specifically biased towards one type of control process. The B-cue trials of the AX-CPT offer an excellent example of proactive control (see Fig. 1 for AX-CPT illustration). During the AX-CPT, subjects make a target response to the probe letter X, *only* when it follows the cue letter A. All other trials in which X is preceded by any letter other than A (collectively referred to as B-cue trials) require a non-target response. B-cue trials require preparation to inhibit a prepotent response due to the high proportion of AX (target) trials, as one must prepare to inhibit the incorrect, but more frequent, response in order to correctly respond to the subsequent probe letter. In contrast, the single trial Stroop task is uncued and can be biased towards reactive control by decreasing the frequency of Incongruent stimuli (i.e., the word RED printed in green ink). Frequent Congruent trials permit the subject to rely more on word reading to respond quickly and accurately; however, when an infrequent Incongruent stimulus is presented, the subject must react quickly to engage control mechanisms to avoid reading the word and making an error [for review see Botvinick et al., 2001]. In the current study, we leverage these task properties by using the AX-CPT task and color naming Stroop task to compare proactive and reactive control processes in patients with schizophrenia.

Behaviorally, we anticipate that patients with schizophrenia will demonstrate specific deficits on the AX-CPT (i.e., reduced accuracy on AX and BX trials with intact performance on AY and BY), as well as reduced accuracy on Incongruent compared to Congruent trials on the Stroop task when compared to healthy controls. Critically, we also predict that schizophrenia participants will show *more pronounced* behavioral deficits on measures of proactive (AX-CPT) compared to reactive (Stroop) control. With regard to neural recruitment, we hypothesize that schizophrenia participants will demonstrate reduced activation of DLPFC during the AX-CPT (Cue B minus Cue A contrast), which loads on proactive control, with relatively intact PFC recruitment during reactive control in the Stroop task (Incongruent minus Congruent contrast) when compared to control subjects. Given previous findings of reduced conflict-related activity in the ACC during the Stroop task (Kerns et al., 2005), we also anticipate that schizophrenia individuals will show lower ACC activity to conflict stimuli compared to controls. Finally, previous literature has identified a relationship between cognitive control impairment and disorganization symptoms (Barch et al., 1999a,b; Woodward et al., 2003; Yoon et al., 2008). We hypothesize that proactive control in particular, as measured by AX-CPT behavioral performance and associated blood oxygenation level dependent (BOLD) activity in DLPFC, will be more strongly associated with schizophrenia disorganization symptoms.

## 2 Material and methods

### 2.1 Participants

Forty-three first episode patients with schizophrenia spectrum disorders (see Table 1 for diagnoses and medication status at time of testing) were recruited along with 54 control subjects. Of the present sample, 16 schizophrenia and 19 control participants were included in a previous study focused only on the AX-CPT (Yoon et al., 2008). Schizophrenia participants were outpatients within one year of their first psychotic episode. All participants were assessed using the Structured Clinical Interview for the DSM-IV-TR [SCID-I/P; First et al., 2002]. Clinical interviews were conducted by clinicians with masters or doctoral degrees trained to high reliability (kappa > .70; range = .70–1.0). Schizophrenia participants were followed longitudinally and diagnoses were confirmed 6 months after ascertainment. Clinical ratings were collected in the schizophrenia sample using the Scale for the Assessment of Negative Symptoms [SANS; Andreasen, 1983], Scale for the Assessment of Positive Symptoms [SAPS; Andreasen, 1984], and Brief Psychiatric Rating Scale [BPRS; Lukoff et al., 1986]. Three schizophrenia participants had missing data on the SAPS and were not included in symptom analyses using those items (e.g., disorganization). Exclusion criteria for both groups included: Wechsler Abbreviated Scale of Intelligence (WASI) IQ score below 70, alcohol or drug dependence or abuse within 3 months before testing, positive urine toxicology screen for illicit drugs, prior head trauma worse than a Grade I concussion, or contraindication to MRI scanning. Healthy controls were excluded for the following *additional* criteria: any lifetime diagnosis of an Axis I or Axis II disorder or any first-degree relatives with a psychotic disorder. Before testing, a detailed description of the study was provided and written informed consent obtained. The study was approved by the University of California, Davis Institutional Review Board. Subjects completed the AX-CPT and Stroop task during one fMRI session, with the order counterbalanced across subjects. All subjects were paid for their participation.

### 2.2 Measures and data analysis

Task parameters and visual depictions of the AX-CPT and Stroop are presented in Fig. 1. Briefly, in the AX-CPT, subjects make a target response (index finger button press) to the probe letter X only if it was preceded by the cue letter A. All cues and non-target probes require non-target responses (middle finger button press). Target sequence trials are frequent and set up a prepotent tendency to make a target response when the probe letter X occurs. As a result, non-target sequence trials where any non-A cue (collectively called B-cues) is presented and followed by a probe letter X, require the most cognitive control. In the Stroop task, stimuli consisted of one of three color words (RED, GREEN, and BLUE) that were written in one of three color inks (red, green, and blue). Stimuli could be Congruent (word and ink match) or Incongruent (word and ink do NOT match). Subjects respond with a button press corresponding to the color of the ink of the word.

AX-CPT accuracy and reaction time were examined using ANOVA with task condition (AX, AY, BX, BY) as a within-subjects factor and diagnosis as a between-subjects factor. Accuracy and reaction times were based upon probe responses, which were only analyzed if the subject responded correctly to the cue. D-prime context (d′-context) (Cohen et al., 1999; Swets and Sewall, 1963) was computed from AX hits and BX false alarms and analyzed using an independent samples t-test. Stroop accuracy and reaction time were analyzed using ANOVA with task (Congruent, Incongruent) as a within-subjects factor and diagnosis as a between-subjects factor. To evaluate whether the degree of group differences depended upon the task, difference scores were calculated for each task [i.e., raw AX percent correct (hits) minus BX percent error (false alarms) and Congruent percent correct minus Incongruent percent error] and evaluated in a 2 × 2 ANOVA. Although d′-context is computed to provide continuity with previous studies of the AX-CPT, these raw difference scores provide a similarly derived measure of specific control processes that can be used to compare the AX-CPT to the Stroop. To address concerns that these raw score difference values may not be directly comparable, z-transformed difference scores were created using the entire sample and also evaluated in a 2 × 2 ANOVA. Group comparisons on measures that violated sphericity assumptions were adjusted using Greenhouse–Geisser and tests in which equal variances were not assumed.

Notably, comparing two tasks and identifying potential differential deficits in a patient group may be confounded by differences in the measurement properties of the tasks (Chapman and Chapman, 1973, 1978). Therefore, estimates of true score variance for each task and trial type in the control group were computed and compared using the methods described by Chapman and Chapman (1978) in which true score variance is the product of the reliability of the test (coefficient alpha) and the variance of the observed scores.

### 2.3 Functional imaging parameters and data analysis

Imaging data were obtained using a 1.5 T General Electric MRI scanner. Coplanar T1-weighted and T2-weighted structural images were acquired prior to each fMRI sequence. For the AX-CPT, T2*-weighted echoplanar imaging (EPI) sessions were acquired with the following settings: TR = 2000-msec, echo time = 40-msec, flip angle = 90°, and field of view = 22 cm. Functional images consisted of 24 contiguous and interleaved 4.0-mm axial slices with a 3.4-mm^2^ in-plane resolution. For the Stroop Task, T2*-weighted EPI sessions were acquired with the following settings: TR = 1500-msec, echo time = 32-msec, flip angle = 90°, and field of view = 22 cm. Functional images consisted of 20 contiguous and interleaved 4.0-mm axial slices with a 3.4-mm^2^ in-plane resolution and extended 80 mm above to 16 mm below the anterior–posterior commissure line. Preprocessing was completed using Statistical Parametric Mapping-8 (SPM8, http://www.fil.ion.ucl.ac.uk/SPM8), including slice timing correction, spatial realignment, spatial normalization to the EPI Montreal Neurological Institute (MNI) template using a rigid-body transformation followed by non-linear warping, and spatial smoothing using a Gaussian 8-mm full-width half-maximum kernel. Individual fMRI runs were removed from the analysis if translational movement exceeded 4-mm or rotational movement exceeded 3° of within-run movement.

Functional imaging analysis was performed for both event-related tasks in SPM8 with multiple regression in the general linear model framework. All task regressors were modeled and only correct responses were included in the reported contrasts. AX-CPT regressors included the A cue, B cue, and each probe (i.e., AX, AY, BX, BY), while Stroop regressors included Congruent and Incongruent trials. Translational and rotational movement data were included as covariates. Group-level random-effect comparisons were performed between groups for the AX-CPT contrast subtracting the A cue from the B cue regressor (AX-CPT B–A contrast) and Stroop task contrast subtracting the Congruent from Incongruent regressor (Stroop I–C contrast). All between-group contrasts were thresholded at the voxel level with p < 0.01 and clusters were considered significant if they survived cluster level FWE correction of p < 0.05. To examine the interaction between task and group, a mixed-model ANOVA was implemented in SPM8 with task as the within-subject factor (AX-CPT B–A contrast and Stroop I–C contrast) and diagnosis as the between-subject factor.

In addition to whole-brain analyses, a priori hypotheses regarding the DLPFC and ACC prompted interrogation of two regions of interest (ROI). The DLPFC ROI consisted of a bilateral Brodmann Area 46 anatomical mask obtained from the Wake Forest University PickAtlas (Maldjian et al., 2003, 2004). The second ROI was obtained using bilateral anterior cingulate regions defined in the Automated Anatomical Labeling (AAL) atlas (Tzourio-Mazoyer et al., 2002), with pre-genual portions manually removed to isolate dorsal ACC. Beta weights for each ROI were extracted and analyzed from the AX-CPT B–A and Stroop I–C contrasts and evaluated in separate 2 × 2 ANOVAs with diagnosis and task as between- and within-subjects factors, respectively. Given the a priori directional hypothesis that patients would show attenuated activity specifically on the Stroop task in the ACC, a one-tailed t-test was used to test for group differences on this measure. Additionally, in order to accurately interpret patterns of activity in the whole-brain group by task analysis, beta weights were extracted post-hoc from regions surviving whole-brain FWE correction.

### 2.4 Correlation analyses with clinical symptomatology

Planned bivariate correlations (two-tailed, alpha set at p < 0.05) were performed between composite symptom measures [disorganization, reality distortion, and poverty symptoms; see Barch et al., 2003] and cognitive/physiological measures (AX-CPT and Stroop accuracy difference scores, DLPFC and ACC beta weights for AX-CPT B–A and Stroop I–C contrasts). Steiger's Z tests (one-tailed) were performed to compare correlations between tasks. Steiger's Z is preferred over Fisher's Z-test as Steiger's Z takes into account the dependency of correlation coefficients that have an index in common (i.e., comparing correlation coefficients between disorganization and an AX-CPT variable to disorganization and a Stroop variable).

## 3 Results

### 3.1 Demographic results

Participant demographic information is presented in Table 1. The groups did not differ significantly on age, gender, handedness, or parental education. Control subjects completed more years of education (t = 3.521, p = 0.001) and had higher estimated IQ (t = 4.724, p < 0.001) than schizophrenia participants.

### 3.2 Behavioral results

See Table 1 for behavioral data. For AX-CPT accuracy, ANOVA revealed significant main effects of diagnosis, F(1, 95) = 7.61, p = 0.007; and condition, F(3, 93) = 48.43, p < 0.001; and a significant diagnosis by condition interaction, F(3, 93) = 3.58, p = 0.017. Bonferroni-corrected (adjusted alpha level of 0.0125 per test) post-hoc comparisons of individual trial types revealed the predicted pattern of performance, with patients showing worse performance on AX (t = 2.77, p = 0.008) and BX (t = 3.29, p = 0.002), but not on AY (t = 0.569, p = 0.571), or BY (t = 1.61, p = 0.112). Independent sample t-test of d′-context scores revealed significantly lower d′-context in patients with schizophrenia (t = 4.22, p < 0.001). ANOVA of reaction time data revealed a significant main effect of diagnosis F(1, 95) = 6.14, p = 0.015; a significant main effect of condition, F(3, 93) = 170.18, p < 0.001; and a trend level diagnosis by condition interaction, F(3, 93) = 2.45, p = 0.068. Bonferroni-corrected (adjusted alpha level of 0.0125 per test) post-hoc reaction time comparisons for each condition revealed a trend towards longer reaction times for schizophrenia patients on BX (t = 2.48, p = 0.015) and BY (t = 2.55, p = 0.013), with no significant differences on AX (t = 2.10, p = 0.039) or AY trials (t = 1.84, p = 0.070).

For Stroop accuracy, ANOVA revealed significant main effects of diagnosis, F(1, 95) = 4.89, p = 0.029; and condition, F(1, 95) = 38.49, p < 0.001; but no significant diagnosis by condition interaction, F(1, 95) = 1.62, p = 0.206. ANOVA of reaction time data revealed a significant main effect of diagnosis, F(1, 95) = 7.24, p = 0.008; a significant main effect of condition, F(1, 95) = 211.02, p < 0.001; but no significant diagnosis by condition interaction, F(1, 95) = 0.13, p = 0.721. In other words, patients demonstrated lower accuracy and longer reaction times overall compared to controls, but the extent of the performance decrement was not dependent upon the trial type.

ANOVA of accuracy difference scores for the AX-CPT (AX hits minus BX false alarms) and Stroop (Congruent correct minus Incongruent incorrect) revealed significant main effects of diagnosis, F(1, 95) = 15.73, p < 0.001; and task, F(1, 95) = 16.56, p < 0.001; and a significant diagnosis by task interaction, F(1, 95) = 6.82, p = 0.010 (Fig. 3a). Z-transformed difference scores were complicated by the presence of outliers. Consequently, a Winsorizing procedure (Dixon, 1960; Hastings et al., 1947) was applied to values exceeding the 99th percentile. ANOVA of these z-transformed difference scores revealed a significant main effect of diagnosis, F(1, 95) = 14.386, p < 0.001; no main effect of task, F(1, 95) = 0.157, p = 0.692; and a significant diagnosis by task interaction, F(1, 95) = 3.97, p < 0.050. While the z-transformed measure paralleled the raw difference score measure in terms of a main effect of group and group by task interaction, the main effect of task was not replicated. These results are encouraging and suggest that when z-transformed, the difference scores reflect comparable levels of cognitive control difficulty overall, but still reveal an interaction such that patients with schizophrenia show a decline in performance in the proactive compared to reactive condition. Given that the z-transformed and raw difference scores showed similar results in the interaction, raw difference scores were used for correlation analyses.

### 3.3 True score variance measurement

Overall, across all trial types, AX-CPT true score variance (σ^2^_T_ = 64.30) was markedly similar to Stroop true score variance (σ^2^_T_ = 69.98). Examining individual trial types for the AX-CPT, AX trials showed the highest true score variance (σ^2^_T_ = 23.86), followed by AY (σ^2^_T_ = 7.58), BX (σ^2^_T_ = 2.74), and BY (σ^2^_T_ = 0.25) trials. For Stroop, Congruent trials (σ^2^_T_ = 22.81) showed slightly higher true score variance than Incongruent trials (σ^2^_T_ = 15.76). Given that our main comparisons of interest involve AX hits versus BX false positives in the AX-CPT and Congruent correct versus Incongruent errors, true score variance values for these trial types are the most critical. While AX and Congruent true score variance estimates are very similar, Incongruent true score variance is over five times larger than BX true score variance. Given that tasks with greater true score variance are more likely to (erroneously) show greater differential deficits, the fact that BX trials show lower true score variance argues against the possibility that AX-CPT specific deficits (and relatively intact Stroop performance) in patients with schizophrenia is due purely to differences in measurement properties of the tasks.

### 3.4 Functional MRI results

#### 3.4.1 Within-group analyses

**Fig. 2:**
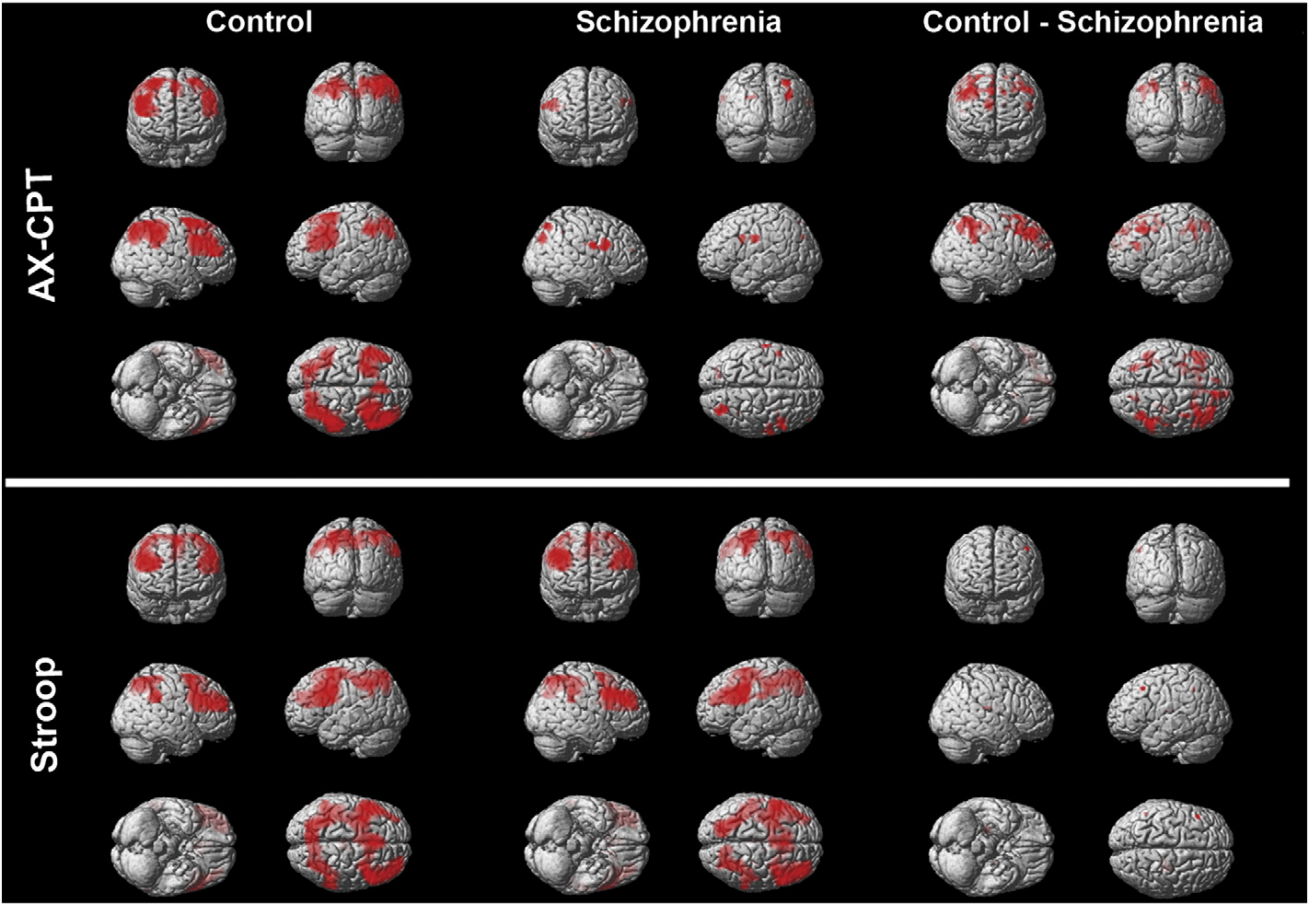
Within- and between-group results for AX-CPT B–A contrast and Stroop I–C contrast at an uncorrected threshold of p < .01 (for display purposes) in healthy control subjects and schizophrenia patients.

Table 2 summarizes data for regions with significant activation, while Fig. 2 illustrates uncorrected whole-brain results. In the AX-CPT B–A contrast, controls showed activation in the predicted network of frontal and parietal regions, including bilateral DLPFC, bilateral parietal cortex, and anterior cingulate/supplementary motor area. In contrast, patients did not demonstrate significant suprathreshold activation in this contrast, although they did show activation across this network at lower, uncorrected thresholds.

In the Stroop I–C contrast, controls showed robust activation in many regions typically activated during cognitive control, including bilateral DLPFC, anterior cingulate/supplementary motor area, and left parietal cortex. Patients showed a similar pattern of activation, including bilateral DLPFC, bilateral parietal cortex, and anterior cingulate/supplementary motor area.

To evaluate the potential impact of medication on brain activity, individuals who were currently taking antipsychotic medication were compared to unmedicated subjects at both a priori regions of interest. There was no significant effect of medication on DLPFC activity during the AX-CPT (t = 1.63, p = 0.120; Cue B minus Cue A), DLPFC activity during the Stroop (t = 0.71, p = 0.483; Incongruent minus Congruent), ACC activity during the AX-CPT (t = 1.58, p = 0.121), or ACC activity during the Stroop (t = 0.32, p = 0.753; Incongruent minus Congruent).

#### 3.4.2 Between-group analyses

In the whole-brain AX-CPT B–A contrast, control subjects demonstrated significantly more activity in right DLPFC as well as right inferior parietal cortex compared to patients. Similarly, control subjects demonstrated significantly more activity in the a priori DLPFC ROI compared to patients with schizophrenia (t = 2.45, p = 0.016; Cue B minus Cue A). No significant differences were found in the a priori structurally defined dorsal ACC ROI for the AX-CPT B–A contrast (t = 1.28, p = 0.205; Cue B minus Cue A). No clusters reached significance in the whole-brain between-group comparison of the Stroop I–C contrast. However, the a priori structurally defined dorsal ACC ROI revealed a trend towards greater activity in controls compared to patients in the primary Stroop contrast (t = 1.42, p = .080; Incongruent minus Congruent). No significant difference was identified in the DLPFC ROI for the Stroop task (t = 0.47, p = 0.643; Incongruent minus Congruent).

#### 3.4.3 Task by group analyses

**Fig. 3:**
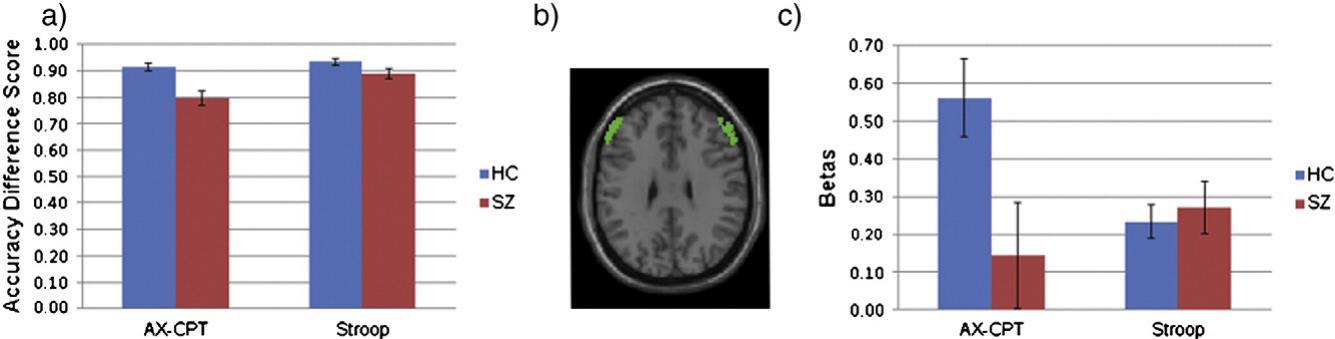
a) Accuracy difference scores representing the group by task interaction, in which patients with schizophrenia demonstrate a greater decrease in performance compared to controls in proactive (AX-CPT = AX Hits minus BX False Alarms) compared to reactive (Stroop = Congruent Hits minus Incongruent Errors) control. b) Horizontal slice view of a priori bilateral DLPFC ROI. c) Beta values (AX-CPT B–A and Stroop I–C) from a priori bilateral DLPFC ROI representing the group by task interaction. Error bars reflect standard error of the mean.

**Fig. 4:**
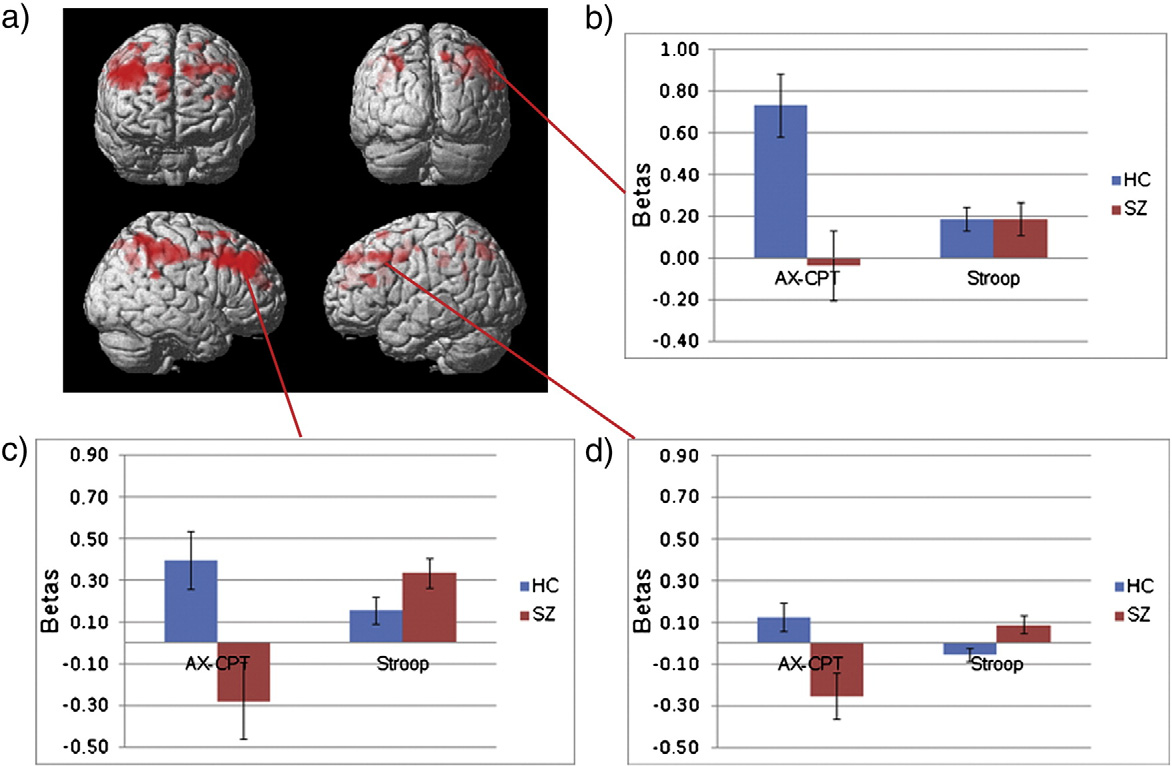
a) Whole-brain thresholded at p < .01 representing the group by task interaction, in which healthy controls (HC) demonstrate greater increases than patients (SZ) in BOLD activity in proactive (AX-CPT B–A) compared to reactive (Stroop I–C) control. The subsequent column graphs represent beta values from the three significant FWE cluster corrected regions representing the group by task interaction: b) inferior parietal cortex, c) right DLPFC, and d) left DLPFC.

The whole-brain task by group interaction analysis revealed significant clusters in bilateral DLPFC and right inferior parietal cortex, with control subjects showing greater increases in activation in the AX-CPT B–A contrast compared to the Stroop I–C contrast, while patients with schizophrenia did not (Fig. 4a). This pattern of results was corroborated in the a priori DLPFC ROI (Fig. 3c), which revealed a significant group by task interaction, F(1, 95) = 6.66, p = 0.011; a trend level main effect of group, F(1, 95) = 3.64, p = 0.059; and no significant main effect of task, F(1, 95) = 1.30, p = 0.257. The a priori ACC ROI revealed no group by task interaction, F(1, 95) = 0.044, p = 0.834; a trend level main effect of group, F(1, 95) = 3.88, p = 0.052; and no significant main effect of task, F(1, 95) = 1.80, p = 0.183. To accurately interpret activity in regions surviving FWE correction in the whole-brain task by group interaction, we also evaluated beta weights extracted from the local maximum coordinates in each significant region (left and right DLPFC and right parietal cortex; see Table 2 for coordinates and Fig. 4 for graphs). Left DLPFC revealed a significant group by task interaction, F(1, 95) = 15.91, p < 0.001; a trend level main effect of group, F(1, 95) = 2.93, p = 0.090; and no significant main effect of task, F(1, 95) = 1.48, p = 0.227. Right DLPFC revealed a significant group by task interaction, F(1, 95) = 10.72, p = 0.001; a main effect of group, F(1, 95) = 4.69, p = 0.033; and no significant main effect of task, F(1, 95) = 2.05, p = 0.155. The right inferior parietal ROI revealed a significant group by task interaction, F(1, 95) = 10.74, p = 0.001; a significant main effect of group, F(1, 95) = 9.05, p = 0.003; and no significant main effect of task, F(1, 95) = 1.89, p = 0.173. In contrast there were no regions in the whole-brain interaction analysis in which patients demonstrated greater increases than controls.

### 3.5 Associations with clinical symptomatology

**Fig. 5:**
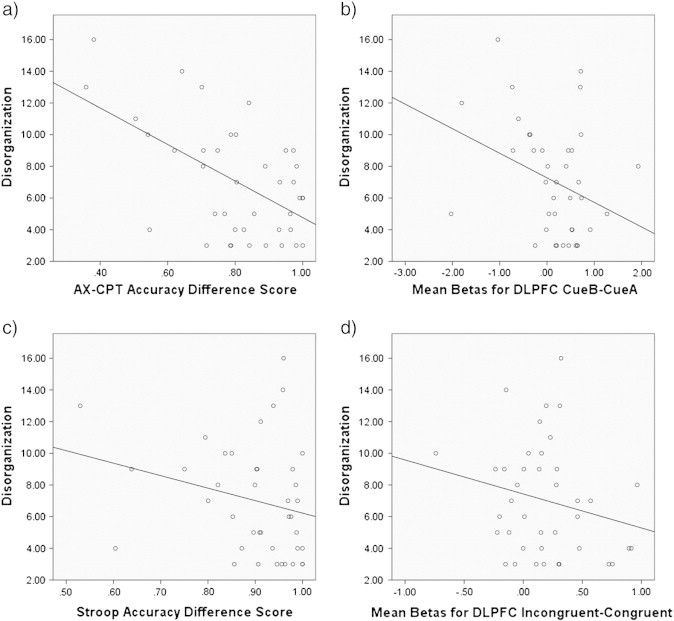
Correlations between disorganization symptom scores in patients with schizophrenia and a) AX-CPT accuracy difference score, b) DLPFC mean beta values during the AX-CPT task (B–A contrast), c) Stroop accuracy difference score, and d) DLPFC mean beta values during the Stroop task (I–C contrast).

Bivariate correlations in the patient group examined the relationship between performance (i.e., AX hits minus BX false alarms and Congruent hits minus Incongruent errors) and DLPFC activity (proactive and reactive). Better AX-CPT performance was significantly associated with higher proactive DLPFC activity (Cue B minus Cue A), r(43) = 0.343, p = 0.025, while Stroop performance was not significantly associated with reactive DLPFC activity (Incongruent minus Congruent), r(43) = − 0.228, p = 0.141. No relationships were found with ACC activity. Lower disorganization symptomatology was associated with a higher AX-CPT accuracy difference score, r(40) = − 0.56, p < 0.001, as well as higher BOLD recruitment in DLPFC during the AX-CPT B–A contrast, r(40) = − 0.33, p = 0.041 (Fig. 5). There was no association between disorganization and the Stroop accuracy difference score or BOLD in DLPFC or ACC during the Stroop (all p > 0.12; Incongruent minus Congruent).

Steiger's Z transformations determined whether disorganization symptoms were more strongly associated with performance and DLPFC activity on the AX-CPT (Cue B minus Cue A) compared to the Stroop (Incongruent minus Congruent). Behavioral performance on the AX-CPT showed a significantly stronger relationship to disorganization, compared to the relationship between Stroop performance and disorganization (p = 0.03). There was not a significant difference in the strength of the relationship between disorganization and DLPFC activity during the AX-CPT compared to the Stroop task (p = 0.28). Notably, the relationship between disorganization and proactive control processes was specific in that correlations with reality distortion, r(42) = − 0.108, p = 0.50, and poverty symptoms, r(43) = − 0.131, p = 0.40, were low.

## 4 Discussion

We used two well-validated cognitive paradigms to probe the neural substrates supporting reactive and proactive control processes in a large sample of patients with first episode schizophrenia. Behaviorally, patients with schizophrenia generally demonstrated worse performance on both tasks compared to controls. However, the extent of the performance decrement was much larger for the proactive control measure examining AX hits and BX false alarms compared to the reactive control measure examining Congruent correct and Incongruent error trials. Neuroimaging results revealed robust activation in both reactive and proactive tasks in healthy controls, represented by lateral prefrontal (BA9, BA46, ACC), and inferior parietal regions during the Stroop I–C contrast and prefrontal (BA9, BA46, ACC) and parietal cortex during the AX-CPT B–A contrast. While patients demonstrated activity comparable to controls in the Stroop, activity was markedly reduced during the AX-CPT, with no activity surviving the cluster-wise threshold. More importantly, the interaction analysis of group and task showed that controls increased activity in DLPFC as well as inferior parietal cortex during proactive compared to reactive control, while patients did not show this increase. Taken together these results suggest that schizophrenia individuals demonstrate relatively preserved engagement of the fronto-parietal network during reactive control, but show a reduced ability to increase recruitment in DLPFC and parietal cortex for proactive control.

Additionally, we identified significant relationships between disorganization and both performance and reduced DLPFC activity during proactive control (AX-CPT B–A contrast). These data corroborate the previous work identifying a relationship between disorganization and DLPFC activity (Edwards et al., 2010) and DLPFC connectivity (Yoon et al., 2008). In contrast, no significant relationships were found between disorganization and any metric of reactive control (e.g., Stroop performance, DLPFC I–C activity, or ACC I–C activity). Furthermore, the relationship between disorganization and AX-CPT performance was stronger than the relationship to Stroop performance, suggesting that disorganization is more strongly associated with proactive control processes. These data, taken in the context of the existing literature, suggest that while reactive control deficits have been identified on the Stroop [for review see Henik and Salo, 2004], proactive control processes may be a more robust link to disorganized clinical symptomatology and underlying neuropathophysiology.

These data shed additional light on the pathophysiology of impaired cognition in schizophrenia in several ways. First, they add to an already substantial literature identifying DLPFC impairment in schizophrenia [see Glahn et al., 2005; Minzenberg et al., 2009 for meta-analytic reviews]. Consistent with recent work (Edwards et al., 2010; Perlstein et al., 2003; Yoon et al., 2008), between-group comparisons revealed significantly reduced DLPFC recruitment during the AX-CPT in schizophrenia individuals. Our findings of adequate recruitment of DLPFC and trend level hypoactivation of ACC during reactive control must be considered in the context of some inconsistency in the literature, with some studies of the Stroop revealing PFC hyperactivation in patients (Weiss et al., 2003) and others identifying PFC hypoactivation (Carter et al., 1997; Yucel et al., 2002). These inconsistencies may partly be the result of variability in task design, with some studies including neutral stimuli, presentation in block- or event-related designs, and modifications of the task in which the subject has to explicitly identify whether the word and color are congruent or not as opposed to identifying the ink color with a response. Stage of illness may also play a role as we have previously reported robust ACC decreases using a similar design to the present study in chronic patients (Kerns et al., 2005). Second, our data provide additional support for the dual mechanisms of control theory proposed by Braver et al. (2007). Notably, these data suggest relatively intact reactive control in patients with schizophrenia and imply that prefrontal control deficits in schizophrenia reflect a stronger loading on proactive control processes, reflected in decreased fronto-parietal recruitment. Reduced recruitment of PFC is consistent with the model we presented in a recent review, which theoretically links cellular abnormalities in the PFC with altered inter-regional cortical connectivity, cognitive control dysfunction, and disorganization (Lesh et al., 2011). Sustained activity in the PFC in non-human primates performing working memory tasks depends upon both dopamine (DA) and norepinephrine (NE), modulating neuronal activity through D1 and alpha 2 adrenergic receptors, respectively (Arnsten and Li, 2005; Arnsten et al., 1988; Brozoski et al., 1979; Cai and Arnsten, 1997). GABA-ergic interneurons are also integral to this process (Gonzalez-Burgos et al., 2010; Lewis et al., 2008). The present data suggest that mechanisms supporting the sustained PFC activity needed for proactive control may be particularly disrupted in schizophrenia. Treatments that remediate these processes, including those that target catecholaminergic and GABA-ergic function in local circuits in this region of the brain, remain promising as interventions to enhance cognitive function in schizophrenia.

One limitation of the current study is the potential effect of medication on fMRI measures. Current evidence suggests that medication effects are not the primary contributor to reduced prefrontal BOLD response during higher-order cognition (Honey et al., 1999; Snitz et al., 2005). Additionally, in the present study, there were no differences between medicated and unmedicated patients in DLPFC and ACC activity across tasks. Finally, since the patients in the present study were very early in the course of illness, it is likely that decreased BOLD response of DLPFC during proactive control is robust and unrelated to the long-term effects of medication exposure or illness chronicity. Another potential limitation is the use of two different tasks, which may have differences in measurement properties that complicate interpretation of a differential deficit. To examine this issue, true score variance values were computed for each task and found to be markedly similar. Furthermore, when examining trial types that were most critical to evaluating proactive and reactive control (i.e., trial types used to compute difference measures), results indicated that Incongruent trials on the Stroop had much higher true score variance than BX trials on the AX-CPT. These data suggest that patients should be more likely to show a performance deficit on the reactive contrast of interest, which contradict our findings of more pronounced proactive deficits, and provide some evidence that our results are not due to differences in measurement properties of the tasks. Finally, differences in scanning parameters between the AX-CPT and Stroop raises the concern that reduced coverage and/or a decreased signal-to-noise ratio (SNR) in the Stroop may have influenced the results. In terms of coverage, within-group results for the Stroop show full coverage of PFC and parietal regions and only voxels that are present in all subjects across both tasks are included in the ANOVA. Differences in the TR and TE raise potential concerns about differences in SNR between the tasks. Although a theoretical SNR can be calculated, it remains difficult to quantify the end result of a lowered TR (which should lower SNR) and a lower TE (which should raise SNR) in the Stroop compared to the AX-CPT. While there may be concerns about decreased SNR in the Stroop, both groups showed robust activation on the Stroop I–C contrast, with peak T-values much higher than the AX-CPT B–A contrast. Additionally, our main findings represent a group by task interaction primarily driven by lower activity in the schizophrenia sample during the AX-CPT with high and relatively comparable activity in the Stroop for both groups. We might anticipate lower SNR in the Stroop to contribute to a main effect of task. However, there was no main effect of task present in the whole-brain ANOVA or any of the a priori or post-hoc ROIs. Consequently, it would be unlikely for these differences in scanning parameters to account for the specific pattern of results we present.

### 4.1 Conclusions and future directions

In summary, the current study contributes to our understanding of cognitive control deficits in schizophrenia by highlighting dissociable processes that provide a more precise understanding of the cognitive and neural mechanisms underlying impaired cognition in the illness. Results suggest that proactive control and associated fronto-parietal dysfunction may represent a more robust marker of disease pathology associated with the clinical presentation of the disorder (i.e., disorganization) in first episode patients. Future studies may leverage these findings by tailoring cognitive training paradigms, neurostimulation and medication development to specifically target proactive control mechanisms in the prefrontal cortex. Future studies should also explore how stage of illness plays a role in the integrity of proactive and reactive control since alterations in elements of reactive control such as the ACC appear more robust in published studies involving chronic patients. Finally, future studies should focus on whether this profile of more pronounced proactive control deficits is also present in other psychiatric populations (i.e., mood disorders and autism) who present with impaired cognitive control (Pompei et al., 2011; Solomon et al., 2009; Strakowski et al., 2005; Wagner et al., 2006).

## Figures and Tables

**Table 1:** Demographic, clinical, and behavioral characteristics for patients with schizophrenia and healthy controls.

Characteristic	Schizophrenia (n = 43)	Healthy control (n = 54)
Years of education: mean ± SD (range)	12.67 ± 1.91 (9–17)	14.09 ± 2.02 (10–20)⁎
Years of parental education: mean ± SD (range)	14.87 ± 2.30 (10–19)	14.28 ± 2.44 (8–20)
Gender: n males (%)	34 (79%)	35 (65%)
Ethnicity: n Caucasian (%)	22 (51%)	28 (52%)
Handedness: n left-handed (%)	0 (0%)	4 (7%)
WASI IQ: mean ± SD (range)	102.12 ± 13.10 (78–131)	113.63 ± 10.67 (85–135)⁎
Diagnosis: n (%)		
Schizophrenia	41 (96%)	–
Schizoaffective	1 (2%)	–
Schizophreniform	1 (2%)	–
Medication status: n (%)		
Unmedicated	15 (35%)	–
Atypical antipsychotic	27 (63%)	–
Typical and atypical antipsychotic	1 (2%)	–
Symptom scores: mean ± SD (range)		
Disorganization symptoms	7.03 ± 3.53 (3–16)	–
Reality distortion symptoms	16.07 ± 6.18 (4–29)	–
Poverty symptoms	13.49 ± 5.69 (3–26)	–
Behavioral data		
AX-CPT accuracy: mean ± SD		
AX	.94 ± .08	.98 ± .03
AY	.82 ± .21	.84 ± .19
BX	.86 ± .14	.94 ± .08
BY	.98 ± .04	.99 ± .03
AX-CPT reaction time: mean (ms) ± SD		
AX	613 ± 184	545 ± 122
AY	774 ± 180	711 ± 148
BX	761 ± 302	625 ± 219
BY	664 ± 224	560 ± 164
Stroop accuracy: mean ± SD		
Congruent	.97 ± .03	.99 ± .02
Incongruent	.92 ± .11	.95 ± .06
Stroop reaction time: mean (ms) ± SD		
Congruent	685 ± 131	622 ± 85
Incongruent	785 ± 158	717 ± 120

SD, standard deviation.

⁎ p < 0.05.

**Table 2:** Regions of significant activation (height p < 0.01; FWE cluster corrected p < 0.05).

		MNI coordinates			
Region	Brodmann's area	x	y	z	T voxel
*AX-CPT B–A contrast*					
Control					
L superior parietal cortex	7	− 50	− 70	50	5.77
R DLPFC	9	52	12	36	5.68
R inferior parietal cortex	40	50	− 44	50	5.63
L DLPFC	46	− 48	32	24	5.42
L inferior parietal cortex	40	− 44	− 52	42	5.32
R superior parietal cortex	7	34	− 68	44	5.27
R DLPFC	46	56	28	28	5.2
R ACC/SMA	32/8	2	22	48	4.27
Schizophrenia					
No significant clusters					
Control > schizophrenia					
R inferior parietal cortex	40	52	− 46	48	4.02
R DLPFC	9/46	54	28	34	3.37
Schizophrenia > control					
No significant clusters					

*Stroop I–C contrast*					
Control					
L DLPFC	9	− 48	10	30	8.85
L inferior parietal cortex	40	− 32	− 58	52	7.23
L ACC/SMA	32/8	− 2	16	58	6.7
L inferior parietal cortex	40	− 38	− 50	50	6.01
R DLPFC	46	44	24	26	6.16
Schizophrenia					
L DLPFC	9	− 48	4	36	7.76
R DLPFC	9	42	12	36	6.77
R DLPFC	46	40	36	28	6.01
L superior parietal cortex	7	− 28	− 58	48	5.7
R superior parietal cortex	7	32	− 60	54	5.48
L ACC/SMA	32/8	− 2	16	52	4.91
Control > schizophrenia					
No significant clusters					
Schizophrenia > control					
No significant clusters					

*AX-CPT B–A > Stroop I–C*					
Control > schizophrenia					
L DLPFC	9	− 22	20	40	4.07
R inferior parietal cortex	40	52	− 46	48	3.78
R DLPFC	9	36	38	38	3.73
Schizophrenia > control					
No significant clusters					

MNI, Montreal Neurological Institute; DLPFC, dorsolateral prefrontal cortex; ACC, anterior cingulate cortex; SMA, supplementary motor area; R, right; L, left.

## References

[bb0005] Andreasen N. (1983). The Scale for the Assessment of Negative Symptoms (SANS).

[bb0010] Andreasen N. (1984). The Scale for the Assessment of Positive Symptoms (SAPS).

[bb0015] Arnsten A.F., Li B.M. (2005). Neurobiology of executive functions: catecholamine influences on prefrontal cortical functions. Biological Psychiatry.

[bb0020] Arnsten A.F., Cai J.X., Goldman-Rakic P.S. (1988). The alpha-2 adrenergic agonist guanfacine improves memory in aged monkeys without sedative or hypotensive side effects: evidence for alpha-2 receptor subtypes. Journal of Neuroscience.

[bb0025] Asaad W.F., Rainer G., Miller E.K. (2000). Task-specific neural activity in the primate prefrontal cortex. Journal of Neurophysiology.

[bb0030] Barch D.M., Ceaser A. (2012). Cognition in schizophrenia: core psychological and neural mechanisms. Trends in Cognitive Sciences.

[bb0035] Barch D.M., Carter C.S., Hachten P.C., Usher M., Cohen J.D. (1999). The “benefits” of distractibility: mechanisms underlying increased Stroop effects in schizophrenia. Schizophrenia Bulletin.

[bb0040] Barch D.M., Carter C.S., Perlstein W., Baird J., Cohen J.D., Schooler N. (1999). Increased stroop facilitation effects in schizophrenia are not due to increased automatic spreading activation. Schizophrenia Research.

[bb0045] Barch D.M., Carter C.S., Braver T.S., Sabb F.W., MacDonald A., Noll D.C., Cohen J.D. (2001). Selective deficits in prefrontal cortex function in medication-naive patients with schizophrenia. Archives of General Psychiatry.

[bb0050] Barch D.M., Carter C.S., MacDonald A.W., Braver T.S., Cohen J.D. (2003). Context-processing deficits in schizophrenia: diagnostic specificity, 4-week course, and relationships to clinical symptoms. Journal of Abnormal Psychology.

[bb0060] Botvinick M.M., Braver T.S., Barch D.M., Carter C.S., Cohen J.D. (2001). Conflict monitoring and cognitive control. Psychological Review.

[bb0065] Braver T.S., Gray J.R., Burgess G.C., Conway A.R.A., Jarrold C., Kane M.J., Miyake A., Towse J. (2007). Explaining the many varieties of working memory variation: dual mechanisms of cognitive control. Variation in Working Memory.

[bb0070] Braver T.S., Paxton J.L., Locke H.S., Barch D.M. (2009). Flexible neural mechanisms of cognitive control within human prefrontal cortex. Proceedings of the National Academy of Sciences of the United States of America.

[bb0075] Brozoski T.J., Brown R.M., Rosvold H.E., Goldman P.S. (1979). Cognitive deficit caused by regional depletion of dopamine in prefrontal cortex of rhesus monkey. Science.

[bb0080] Cai J.X., Arnsten A.F. (1997). Dose-dependent effects of the dopamine D1 receptor agonists A77636 or SKF81297 on spatial working memory in aged monkeys. Journal of Pharmacology and Experimental Therapeutics.

[bb0085] Carter C.S., Mintun M., Nichols T., Cohen J.D. (1997). Anterior cingulate gyrus dysfunction and selective attention deficits in schizophrenia: [15O]H2O PET study during single-trial Stroop task performance. The American Journal of Psychiatry.

[bb0090] Chapman L.J., Chapman J.P. (1973). Problems in the measurement of cognitive deficit. Psychological Bulletin.

[bb0095] Chapman L.J., Chapman J.P. (1978). The measurement of differential deficit. Journal of Psychiatric Research.

[bb0100] Cohen J.D., Servan-Schreiber D. (1992). Context, cortex, and dopamine: a connectionist approach to behavior and biology in schizophrenia. Psychological Review.

[bb0105] Cohen J.D., Braver T.S., O'Reilly R.C. (1996). A computational approach to prefrontal cortex, cognitive control and schizophrenia: recent developments and current challenges. Philosophical Transactions of the Royal Society of London. Series B, Biological Sciences.

[bb0110] Cohen J.D., Barch D.M., Carter C., Servan-Schreiber D. (1999). Context-processing deficits in schizophrenia: converging evidence from three theoretically motivated cognitive tasks. Journal of Abnormal Psychology.

[bb0115] Dixon W.J. (1960). Simplified estimation from censored normal samples. Annals of Mathematical Statistics.

[bb0120] Edwards B.G., Barch D.M., Braver T.S. (2010). Improving prefrontal cortex function in schizophrenia through focused training of cognitive control. Frontiers in Human Neuroscience.

[bb0125] Egner T., Hirsch J. (2005). Cognitive control mechanisms resolve conflict through cortical amplification of task-relevant information. Nature Neuroscience.

[bb0130] First M.B., Spitzer R.L., Gibbon M., Williams J.B.W. (2002). Structured Clinical Interview for DSM-IV-TR Axis I Disorders, Research Version, Patient Edition. (SCID-I/P).

[bb0135] Glahn D.C., Ragland J.D., Abramoff A., Barrett J., Laird A.R., Bearden C.E., Velligan D.I. (2005). Beyond hypofrontality: a quantitative meta-analysis of functional neuroimaging studies of working memory in schizophrenia. Human Brain Mapping.

[bb0140] Goldman-Rakic P.S. (1995). Cellular basis of working memory. Neuron.

[bb0145] Gonzalez-Burgos G., Hashimoto T., Lewis D.A. (2010). Alterations of cortical GABA neurons and network oscillations in schizophrenia. Current Psychiatry Reports.

[bb0150] Hastings C., Mosteller F., Tukey J.W., Winsor C.P. (1947). Low moments for small samples: a comparative study of order statistics. Annals of Mathematical Statistics.

[bb0155] Henik A., Salo R. (2004). Schizophrenia and the stroop effect. Behavioral and Cognitive Neuroscience Reviews.

[bb0160] Honey G.D., Bullmore E.T., Soni W., Varatheesan M., Williams S.C., Sharma T. (1999). Differences in frontal cortical activation by a working memory task after substitution of risperidone for typical antipsychotic drugs in patients with schizophrenia. Proceedings of the National Academy of Sciences of the United States of America.

[bb0165] Kerns J.G., Cohen J.D., MacDonald A.W., Johnson M.K., Stenger V.A., Aizenstein H., Carter C.S. (2005). Decreased conflict- and error-related activity in the anterior cingulate cortex in subjects with schizophrenia. The American Journal of Psychiatry.

[bb0170] Lesh T.A., Niendam T.A., Minzenberg M.J., Carter C.S. (2011). Cognitive control deficits in schizophrenia: mechanisms and meaning. Neuropsychopharmacology.

[bb0175] Lewis D.A., Cho R.Y., Carter C.S., Eklund K., Forster S., Kelly M.A., Montrose D. (2008). Subunit-selective modulation of GABA type A receptor neurotransmission and cognition in schizophrenia. The American Journal of Psychiatry.

[bb0180] Lukoff D., Nuechterlein K.H., Ventura J. (1986). Manual for the Expanded Brief Psychiatric Rating Scale (BPRS). Schizophrenia Bulletin.

[bb0185] MacDonald A.W., Carter C.S. (2003). Event-related FMRI study of context processing in dorsolateral prefrontal cortex of patients with schizophrenia. Journal of Abnormal Psychology.

[bb0190] MacDonald A.W., Cohen J.D., Stenger V.A., Carter C.S. (2000). Dissociating the role of the dorsolateral prefrontal and anterior cingulate cortex in cognitive control. Science.

[bb0195] Maldjian J.A., Laurienti P.J., Kraft R.A., Burdette J.H. (2003). An automated method for neuroanatomic and cytoarchitectonic atlas-based interrogation of fMRI data sets. NeuroImage.

[bb0200] Maldjian J.A., Laurienti P.J., Burdette J.H. (2004). Precentral gyrus discrepancy in electronic versions of the Talairach atlas. NeuroImage.

[bb0205] Miller E.K., Cohen J.D. (2001). An integrative theory of prefrontal cortex function. Annual Review of Neuroscience.

[bb0210] Minzenberg M.J., Laird A.R., Thelen S., Carter C.S., Glahn D.C. (2009). Meta-analysis of 41 functional neuroimaging studies of executive function in schizophrenia. Archives of General Psychiatry.

[bb0215] Perlstein W.M., Dixit N.K., Carter C.S., Noll D.C., Cohen J.D. (2003). Prefrontal cortex dysfunction mediates deficits in working memory and prepotent responding in schizophrenia. Biological Psychiatry.

[bb0220] Pompei F., Jogia J., Tatarelli R., Girardi P., Rubia K., Kumari V., Frangou S. (2011). Familial and disease specific abnormalities in the neural correlates of the Stroop Task in Bipolar Disorder. NeuroImage.

[bb0225] Servan-Schreiber D., Cohen J.D., Steingard S. (1996). Schizophrenic deficits in the processing of context. A test of a theoretical model. Archives of General Psychiatry.

[bb0230] Snitz B.E., MacDonald A., Cohen J.D., Cho R.Y., Becker T., Carter C.S. (2005). Lateral and medial hypofrontality in first-episode schizophrenia: functional activity in a medication-naive state and effects of short-term atypical antipsychotic treatment. The American Journal of Psychiatry.

[bb0235] Solomon M., Ozonoff S.J., Ursu S., Ravizza S., Cummings N., Ly S., Carter C.S. (2009). The neural substrates of cognitive control deficits in autism spectrum disorders. Neuropsychologia.

[bb0240] Strakowski S.M., Delbello M.P., Adler C.M. (2005). The functional neuroanatomy of bipolar disorder: a review of neuroimaging findings. Molecular Psychiatry.

[bb0245] Swets J.A., Sewall S.T. (1963). Invariance of signal detectability over stages of practice and levels of motivation. Journal of Experimental Psychology.

[bb0250] Tzourio-Mazoyer N., Landeau B., Papathanassiou D., Crivello F., Etard O., Delcroix N., Mazoyer B., Joliot M. (2002). Automated anatomical labeling of activations in SPM using a macroscopic anatomical parcellation of the MNI MRI single-subject brain. NeuroImage.

[bb0255] Wagner G., Sinsel E., Sobanski T., Kohler S., Marinou V., Mentzel H.J., Sauer H., Schlosser R.G. (2006). Cortical inefficiency in patients with unipolar depression: an event-related fMRI study with the Stroop task. Biological Psychiatry.

[bb0260] Watanabe M. (1990). Prefrontal unit activity during associative learning in the monkey. Experimental Brain Research.

[bb0265] Watanabe M. (1992). Frontal units of the monkey coding the associative significance of visual and auditory stimuli. Experimental Brain Research.

[bb0270] Weiss E.M., Golaszewski S., Mottaghy F.M., Hofer A., Hausmann A., Kemmler G., Kremser C., Brinkhoff C., Felber S.R., Fleischhacker W.W. (2003). Brain activation patterns during a selective attention test-a functional MRI study in healthy volunteers and patients with schizophrenia. Psychiatry Research.

[bb0275] Woodward T.S., Ruff C.C., Thornton A.E., Moritz S., Liddle P.F. (2003). Methodological considerations regarding the association of Stroop and verbal fluency performance with the symptoms of schizophrenia. Schizophrenia Research.

[bb0280] Yoon J.H., Minzenberg M.J., Ursu S., Walter R., Wendelken C., Ragland J.D., Carter C.S. (2008). Association of dorsolateral prefrontal cortex dysfunction with disrupted coordinated brain activity in schizophrenia: relationship with impaired cognition, behavioral disorganization, and global function. The American Journal of Psychiatry.

[bb0285] Yucel M., Pantelis C., Stuart G.W., Wood S.J., Maruff P., Velakoulis D., Pipingas A., Crowe S.F., Tochon-Danguy H.J., Egan G.F. (2002). Anterior cingulate activation during Stroop task performance: a PET to MRI coregistration study of individual patients with schizophrenia. The American Journal of Psychiatry.

